# Reported oral and anal sex among adolescents and adults reporting heterosexual sex in sub-Saharan Africa: a systematic review

**DOI:** 10.1186/s12978-019-0722-9

**Published:** 2019-05-06

**Authors:** Imran O. Morhason-Bello, Severin Kabakama, Kathy Baisley, Suzanna C. Francis, Deborah Watson-Jones

**Affiliations:** 1 Clinical Research Department, Faculty of Infectious and Tropical Diseases, London School of Hygiene and Tropical Medicine, Keppel St, London, WC1E 7HT UK; 2 Obstetrics and Gynaecology Department, Faculty of Clinical Sciences, College of Medicine, University of Ibadan, Ibadan, Nigeria; 3 Mwanza Intervention Trials Unit, National Institute for Medical Research, PO Box 11936, Mwanza, Tanzania; 4 Department of Infectious Disease Epidemiology, Faculty of Epidemiology and Population Health, London School of Hygiene and Tropical Medicine, Keppel St, London, WC1E 7HT UK

**Keywords:** Oral/anal sex, Sexual behaviour, Heterosexual, Adolescent, Adult, Sub-Saharan Africa

## Abstract

**Background:**

Oral and anal sexual behaviours are increasingly reported among adolescents and adults reporting heterosexual sex in peer-reviewed journals in high income countries, but less is known about these behaviours in low and middle-income countries, especially in sub-Saharan Africa. The aim of this systematic review is to describe the prevalence of, and motivations for, oral and anal sex among adolescents and adults reporting heterosexual sex in sub-Saharan Africa.

**Methods:**

A systematic review of published articles that reported oral and or anal sex in sub-Saharan Africa was conducted from seven databases up to and including 30th August 2018.

**Results:**

Of 13,592 articles, 103 met the inclusion criteria. The prevalence of reporting ever practising oral sex among adolescents, university students and a combined population of adolescents/adults ranged from 1.7–26.6%, 5.0–46.4% and 3.0–47.2% respectively. Similarly, prevalences of reported ever practising anal sex ranged from 6.4–12.4% among adolescents, 0.3–46.5% among university students and 4.3–37.8% amongst combined population of adolescents and adults. Higher prevalences of oral and anal sex were reported among populations at high-risk for sexually transmitted infections and HIV and university students and, in most studies, both behaviours were more commonly reported by males than females. Heterosexual oral and anal sexual acts were associated with some high-risk behaviours such as inconsistent condom use and multiple sexual partners.

**Conclusion:**

Reported oral and anal sex between men and women are prevalent behaviours in sub-Saharan Africa. Health professionals and policy makers should be aware of these behaviours and their potential associated health risks.

**Electronic supplementary material:**

The online version of this article (10.1186/s12978-019-0722-9) contains supplementary material, which is available to authorized users.

## Plain English summary

Oral and anal sexual acts are increasingly reported in peer reviewed journals, especially among adolescents and young adults in high income countries. These behaviours are associated with negative health outcomes such as sexually transmitted infections (STIs). Oral and anal sex may be important unrecognised modes of transmission for STIs, contributing to onward transmission. In addition, STIs in the oropharynx and anus may result in poor health outcomes such as oral and anal cancers; however, oral and anal sex are not always regarded as ‘hetero-normative sexual intercourse’, and are often disregarded by researchers, programmers and policy makers. Importantly, both sexual acts are sometimes perceived by some people to be safer than vaginal sex against pregnancy and STIs, and are associated with lower reported use of condoms to prevent HIV and STIs.

We conducted a systematic review of published scientific papers reporting these behaviours in sub-Saharan Africa between 1946 and 30th August 2018. We investigated the prevalences of oral and anal sex, and factors that influenced these behaviours.

Our findings showed that oral and anal sex were commonly reported among adolescents and adults as well as female sex workers. We found that more boys/men reported oral and anal sex than girls/women in most of the studies, and that a substantial number of those engaging in oral and anal sex did not use barrier methods during those sexual acts.

In summary, oral and anal sexual behaviours are commonly reported in sub-Saharan Africa among people reporting heterosexual sex. While testing for oropharyngeal and anal infections may not be feasible in resource-constrained settings, these data are important for researchers, programmers and policy makers to raise awareness of these potential modes of STI transmission. Information about the risk of STI transmission for oral and anal sex should be included in information, education and counseling programmes for both general and key populations at risk for STIs.

## Background

Condomless heterosexual oral and anal intercourse have been associated with extragenital sexually transmitted infections (STI) such as *Chlamydia trachomatis*, *Neisseria gonorrhoea*, syphilis, hepatitis, herpes simplex virus (HSV) and human papillomavirus (HPV) infections in the anal and oropharyngeal niches [1–6]. Although oral and anal STIs are frequently asymptomatic, they remain an important source of onward transmission [1–3, 5]. Clinical sequelae of oral and anal STIs include pain, anal discharge, ulcerative proctitis, HPV-associated premalignant lesions and cancers [2, 3, 6, 7]. The comparative risk of HIV infection transmission between condomless anal sex and vaginal sex is higher than oral sex, and also, the risk is higher among those engaging in receptive anal sex than insertive anal sex when other HIV prevention methods such as anti-retroviral treatment or preexposure prophylaxis are not used [8, 9].

Several studies have reported a higher prevalence of oral and penile-anal sex among key affected populations’ such as female sex workers (FSWs) [10, 11], men who have sex with men (MSM) [12], entertainment outlet workers, long distance drivers and people who inject drugs, compared to general populations [13]. Most studies report low or inconsistent condom use during oral and anal sex. For example, in Lima, Peru (2010), 98.4% of FSWs aged 18–26 years had performed oral sex in their lifetime and only 20.0% reported condom use during the sexual act [11]. Another study in Peru (2013) showed that 21.2% of FSWs performed oral sex with clients in the previous month, and only 37.6% used condoms consistently while performing oral sex [14]. A study conducted in India (2009–2010) reported that 12.3% of 18–60 year old FSWs engaged in receptive penile-anal intercourse in the past 6 months, and only 48.4% used condoms consistently [15]. In the Netherlands (2016), the prevalence of anal sex in the past 6 months among FSWs aged 18 years and above was 20.0%, and only 31.0% of these FSWs always used condoms with clients [16]. In the USA, a systematic review of anal sex that included published articles between 1987 and 2013 reported that the prevalence of anal sex among FSWs ranged between 0 and 18.0% and that 14.0–82.0% of these FSWs always used condoms during anal sex [17].

Oral and anal sexual behaviours are increasingly reported among general population adolescents and adults reporting heterosexual sex in both developed and developing countries [18–21]. For example, a recent systematic review among young people aged less than 25 years worldwide showed that the average life time prevalence of reported heterosexual anal sex was 22.0%, and this behaviour accounted for 3.0–24.0% of all reported sexual acts [22]. Three sexual behaviour surveys conducted between 1990 and 2012 among men and women aged 16–44 years in the United Kingdom showed that the prevalence of reported heterosexual penetrative anal sex by men in the past year increased from 7.0% in the 1990–1991 survey to 12.2% in the 1999–2001 survey to 17.0% in 2010–2012 [21]. Similarly, the proportion of women that reported any receptive anal sex in the past year also increased from 6.5 to 11.3% to 15.1% over the same periods [21]. Between the first and last surveys, the proportion of those giving or receiving oral sex in the preceding year increased from 65.6 to 75.0% among women and from 69.7 to 77.1% among men [21]. National surveys in Australia in 2001–2002 (aged 16–59 years) and 2012–2013 (aged 16–69 years) also showed a moderate increase in prevalence of reported oral and penile-anal sex over time in both genders [18, 23].

In sub-Saharan Africa (SSA), many studies reporting sexual behaviours in heterosexual relationships have focused on penile-vaginal sex and associated negative health outcomes [24–27]. This has influenced sexual health policies and programmes in many countries within the region [28, 29]. The role of heterosexual oral and anal sexual acts within the spectrum of sexual behaviours needs to be documented within the region, in order to appreciate their potential impact on STI transmission and other associated morbidities such as oral and anal cancers. We conducted a systematic review of the prevalence of, and motivations for, practising heterosexual oral and penile-anal sex, and the cultural interpretations of these behaviours in SSA.

## Methods

This review was conducted in accordance with Preferred Items for Reporting of Systematic Reviews and Meta-analyses (PRISMA) and Meta-analysis of Observational Studies in Epidemiology (MOOSE) guidelines [30, 31]. The protocol was registered in PROSPERO database with registration number CRD42015025311 [32].

### Search strategy

The search was conducted in English using seven databases: Medline; Embase; African-Wide Information; Cinahl; Global Health; Scopus; and Popline databases. We used medical subject headings (MeSH) and text words related to oral and anal sex for the search. The terms used for oral sex were oral (sex OR sexual behaviour OR sexual practices), cunnilingus, oral vaginal contact, fellatio, oral penile contact, anilingus, and oral anal contact. The search terms for anal sex included anal (anus OR anal cavity) sex OR anal (sexual behaviour OR sexual practice) or ano-genital (sex OR intercourse). The search was restricted to SSA by using “AND” before adding different search terms for sub-regions (West Africa OR East Africa OR Central Africa OR Southern Africa), and by specific country names. Multi-continent studies that had separate data from any country in SSA were also considered. The search included published articles from 1946 up to and including the final search of 30th August 2018. We also conducted manual searches of bibiliography of relevant publications on the subject. All titles retrieved from the search were compiled and reviewed with Endnote X 8.0 (Thompson Reuters) by one author (IMB); duplications were removed using the Endnote automated system and through a manual check.

### Eligibility criteria

The eligibility criteria were determined *apriori* in the registered protocol [32], and only published original research articles that reported on oral or anal sex with a partner of the opposite sex in adolescents and adults in SSA were considered. The review excluded articles that focused exclusively on non-consensual heterosexual intercourse and MSM, even if men reported sex with both men and women. Commentaries or review articles, letter to editors, editorials, case series and case reports were also excluded. Oral sex was defined as oral contact with the vulva and or vagina (cunnilingus) or penile shaft (fellatio) or anus (analingus). Anal sex was defined as penetration (insertion) of a man’s penis into the woman’s anus or, for women, reception of the penile shaft into the the woman’s anus.

Two authors (IMB, SK) independently screened the titles and abstracts using the eligibility criteria. Thereafter, full-text of selected articles were independently reviewed again by IMB and SK. Discrepancies at each stage of review were resolved through discussion and consensus. DWJ and SCF served as arbitrators for cases that could not be resolved by discussion.

### Data extraction

Data were extracted by IMB into pre-specified data extraction form prepared in Microsoft Excel 2010, and verified by SK. The data extracted included author and journals’ name, sampling methods, study location, definition of oral and anal sex, prevalence/proportion of those that reported oral and anal sex, including reasons/motivations and risk factors associated with these behaviours. Prevalence was defined as the proportion of those that reported oral/anal sex by the total number of individuals in the study population.

For reporting periods, studies that used “ever had” or “ever experienced” or “life-time experience” for oral and or anal sex were classified as “ever practiced”. Other specific look back reporting periods recorded were “past 12 months”, “past 3 months”, “past 1 month”, “last sexual act” and the “first sexual act”. We classified studies that used any form of random sampling as “probability sampling” while others that used non-probability techniques were categorised as “convenience”or “snowball” or “venue-based” or “volunteer” sampling. Studies that had participants with increased risk of STI were categorised as key affected populations (e.g. FSWs, HIV positive men and women, recreational facility workers such as bar and guesthouse workers, long distance truck drivers and participants described as “high-risk” in the methods sections of eligible publications).

### Assessment of quality of eligible studies

Separate risk of bias tools were used to assess the quality of papers reporting quantitative and qualitative data. For papers reporting quantitative data, a validated tool for observational studies was modified (Additional file 1: Figure S3) [33] by developing a list of methodological features of the eligible studies that could bias the prevalence and risk factor estimates for oral and anal sex. For each study, we assessed documentation of the following items to classify the study as being either at lower or higher risk of bias: description of study population (Yes/No); type of sampling techniques (probability sampling [Yes] or non-probability sampling/not reported [No]); response rate (Yes/No); eligibility criteria (Yes/No); definition of oral sex (Yes/No); definition of anal sex (Yes/No); sexual behaviour roles reported as giving/insertive or receptive/received (Yes/No); risk factor estimates controlled for potential confounders (Yes/No); and inclusion of a statement on the ethical approval (Yes/No). For papers reporting qualitative data, a critical appraisal skill programme tool was used for qualitative studies (Additional file 1: Figure S4) [34]. Each tool has ten fields for assessment; studies with five or more “Yes” fields were considered to be of lower risk of bias.

### Data synthesis

Due to the heterogeneity in study populations, study design, sampling strategy and definitions of exposure of interest in the eligible studies, we performed a descriptive analysis of both quantitative and qualitative studies without providing a pooled estimates by meta-analysis. In the descriptive analysis, data were disaggregated by exposure of interest (oral sex, anal sex or both), gender, population category (key affected or general population), country locations and regions. We presented prevalences of outcomes (oral and anal sex) and risk factors for oral and anal sex in quantitative studies. We also used Minitab 18.0 statistical software (Minitab, Inc.) to graphically present individual value plot of prevalences of oral and anal sex by sub-region, study population, population category and risk of bias to visualise the range in prevalences compared by population type, geographical area and risk of bias. Key findings from qualitative studies were summarised and categorised into the following themes: cultural meaning; interpretations; and reported personal experiences.

## Results

**Fig. 1:**
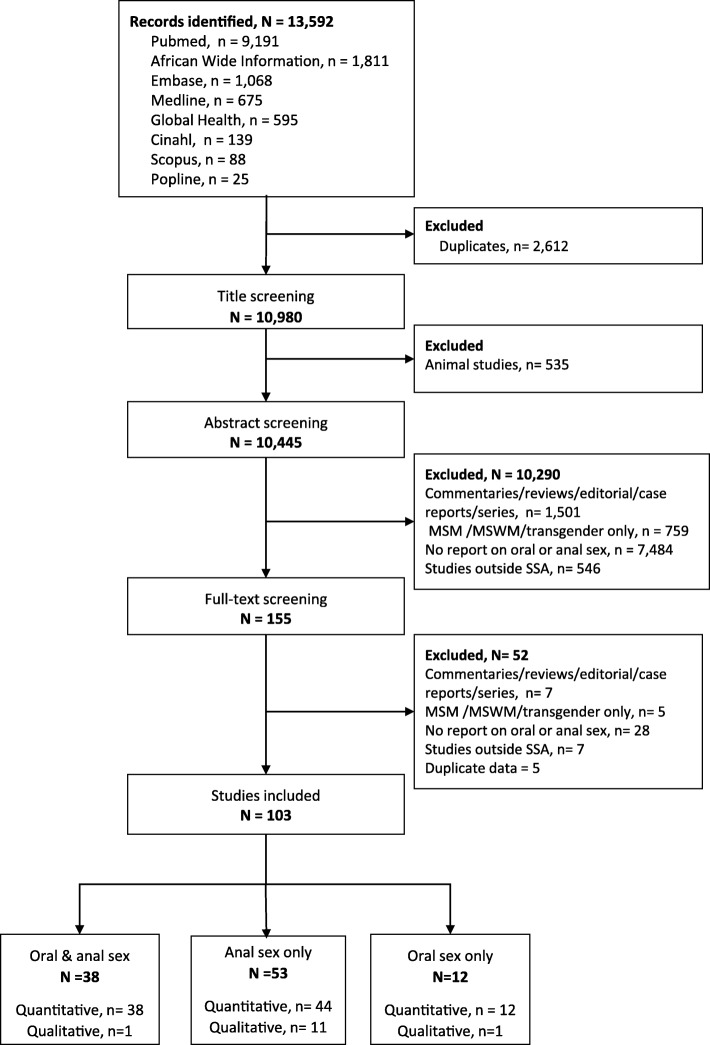
PRISMA Flow for the systematic review

Out of the 13,592 articles retrieved, 155 full texts were reviewed and 103 were included in the descriptive analysis of heterosexual oral and or anal sexual behaviours (Fig. 1). Among the 103 articles reviewed, 38 reported on both oral and anal sex [35–73], 53 reported on anal sex only [74–125], and 12 reported on oral sex only [126–137]. One Nigerian publication out of the 38 articles reported the prevalence of oral and anal sex as a combined outcome [43]. Six articles were mixed method studies (a study each from Ethiopia, Kenya, Nigeria, Tanzania, Zambia and another was conducted in Kenya and Rwanda) [39, 66, 70, 90, 120, 133]. Fifty-nine studies were from Southern Africa, 38 were from East Africa, 20 were from West Africa and four were from Central Africa (Additional file 2: Table S1).

Only nine quantitative studies presented the operational definitions of reported oral sex [37, 38, 52, 54, 66, 71–73, 137] and six studies presented operational definitions of reported anal sex [38, 52, 54, 66, 73, 90] in the methods section of the papers. For oral sex, definitions included oral stimulation of either the external female genitalia (i.e. clitoris, vulva and vagina) or the penis [37]; ejaculation during oral stimulation of the male genitalia [38]; contact between the mouth and penis or vagina or anus [54]; and putting one’s mouth on their partner’s penis or vagina or letting their partner put his or her mouth on one’s penis or vagina [52]. For anal sex, definitions included were ejaculation during anal sex [38]; introduction of the penis into the anus or back passage of the partner [54, 66, 73, 90]; and putting the penis into the partner’s anus or letting the partner insert his penis to her anus [52].

Twelve out of 13 articles presented qualitative data investigating motivations, cultural interpretations and personal experiences of anal sex [66, 90, 100–104, 120, 122–125] (Table 1). Of these, seven studies (two from South Africa [100, 101] and three from Tanzania [103, 104, 124], and two multi-country studies conducted in Kenya, Tanzania and Uganda [102] and Uganda, South Africa and Zimbabwe [123]) used qualitative methods only. Six qualitative studies each on anal sex were conducted among key affected populations [90, 102–104, 124, 133] and general populations [66, 100, 101, 120, 122, 123]. No studies included adolescents. One article reported on perception of FSWs on the use of condom for oral sex [133].

**Table 1:** Selected data from qualitative studies reporting on heterosexual oral and anal sex in sub-Saharan Africa by year of publication

Author; Year	Country	Study design	Sampling; Data collection methods	Study population	Gender (M/F); Age (yrs)	Summary of Key findings on oral sex	Assessment of risk of bias
Gathece et al. 2000 [133]	Kenya	Mixed methods	Purposive sampling; NS Focus group discussions and In-depth interviews	FSW (KAP)	F: 30.5	Participants said that there was no need to use condom during oral sex because they felt that oral sex is “safer” than vaginal sex.	high-risk of bias
Author; Year	Country	Study design	Sampling; Data collection methods	Study population	Gender (M/F); Age (yrs)	Summary of Key findings on Anal sex	Assessment of risk of bias
Stadler et al. 2007 [100]	South Africa	Qualitative	Purposive sampling; Focus group discussion	Adult women in community (GP)	F; NS	Some of the local terminologies of anal sex that were mentioned by participants included: “pata pata”, “matanyula” and “dog style”. Participants had open discussions on anal sex. They mentioned pornographic films and television as sources of information of anal sex. The reported reasons for engaging in anal sex that were discussed included: a form of partner punishment and coercion, sexual experimentation and partner desire or pleasure. Some of the misconceptions that emerged during discussion were: anal sex is safer than penile-vaginal sex, anal sex was regarded as a form of contraception and it could prevent STIs/HIVs.	low risk of bias
Mavhu et al. 2008 [122]	Zimbabwe	Qualitative	Purposive sampling; 65 In-depth interviews	Adolescent and Adult in the community (GP)	M and F:18–40	Participants were uncomfortable with “anal sex” as a question. They preferred anal sex to be referred to as “sex at a place where wastes (faeces) comes out” or “sex at backside”. Some referred to anal sex as “doggy style”. They described anal sex as “homosexual do”.	low risk of bias
Ndinda et al. 2008 [101]	South Africa	Qualitative	Purposive sampling; 11 Focus group discussions	Adult men and women in community (GP)	MandF; NS	Participants were reluctant to talk about anal sex. They largely used proxy names in Zulu to describe anal sex. There was poor understanding about the meaning of anal sex. They expressed shock and disbelief when the facilitator told them the meaning of anal sex as “having sex in the faecal passage”. Some participants felt that anal sex is practiced only by MSM. They expressed shock and disbelief when the facilitator told them the meaning of anal sex as “having sex in the feacal passage”. Some participant felt that anal sex is practiced only by MSM.	low risk of bias
Ambaw et al. 2010 [66]	Ethiopia	Mixed methods	Purposive sampling; 6 Focus group discussions	University students (GP)	M and F: 17–45	Participants interpreted sexual intercourse to mean heterosexual and vaginal sex. They believed only “whites” (ferenges) accept anal sex as a form of sexual intercourse	low risk of bias
Veldhuijzen et al. 2011 [90]	Kenya and Rwanda	Mixed methods	Purposive sampling; 7 Focus group discussions and 4 In-depth interviews	FSW in Kigali only (KAP)	F; 22–30	Some FSWs had strong negative perceptions about clients requesting anal sex. They believed that anal sex was mostly requested by non-Rwandans. The most common reported reason for engaging in anal sex was a financial incentive. Some participants said that anal sex practices are associated with alcohol use. Some FSWs reported that their clients do not use condoms for anal sex.	low risk of bias
Duby et al. 2014 [102]	Kenya, Tanzania and Uganda	Qualitative	Purposive sampling; 40 Focus group discussions and 54 In-depth interviews	Adult men and women in community/clinics (KAP)	M and F; NS	Participants demonstrated a good knowledge of anal sex, but they were reluctant to discuss their personal experiences. They also regarded anal sex as a taboo. Reported reasons for engaging in anal sex that were discussed included: preserving virginity, contraception, financial incentives, as an alternative when vaginal sex was not feasible (e.g. pregnancy and menstruation), to satisfy their male partner’s pleasure, adventure and novelty. Participants had the misconception that anal sex is safer than vaginal sex, is associated with lesser risk of HIV/STIs and hence there is no reason to use condoms.	low risk of bias
Beckham et al. 2015 [103]	Tanzania	Qualitative	Purposive sampling; 3 Focus group discussions and 30 In-depth interviews	FSW (KAP)	F; 20–40	Participants had open discussion about anal sex including their personal experiences with clients. The main reported reason for engaging anal sex was receiving higher financial incentives than for penile-vaginal sex. Clients paid more for requesting unprotected anal sex.	low risk of bias
Mtenga et al. 2015 [124]	Tanzania	Qualitative	Purposive sampling; 24 Focus group discussions and 81 In-depth interviews	Adult men and women in community (KAP)	M and F; NS	Truck drivers and their female partners said heterosexual anal sex is increasingly being practiced in Tanzania. Reasons for engaging in anal sex by men included: seeking better sexual pleasure than vaginal sex, to avoid pregnancy, to prove to their female partners that they are sexually strong and for sexual adventure. Women mentioned receiving greater financial gains with anal sex compared to vaginal sex, that anal sex helped to retain their sexual partners, that it could prevent pregnancy, was an alternative during menstruation and fattening their buttocks to improve their beauty to suitors as reasons for practicing anal sex. Both men and women said a barrier method is not needed during anal sex because the sexual act is safer than vaginal sex, sexual pleasure will be reduced and there is a fear of losing the condom inside the anus.	low risk of bias
Wamoyi et al. 2015 [104]	Tanzania	Qualitative	Purposive sampling; 4 Focus group discussions and 16 In-depth interviews	Adult men and women in community/clinics (KAP)	MandF; 20–30	There was culture of silence about anal sex and participants used proxy names for the practice. Participants believed that anal sex was mostly practiced by foreigners, young people, FSWs and Arabs. Reported reasons for engaging in anal sex that were described by participants included: adventure, ‘trending’, financial incentives and male partner desire. Women felt that anal sex was associated with delivery complications, and anal incontinence. Male participants reported that engaging in anal sex could cause blockage of the anus and urinary system, and cancer. Both male and female participants believed that anal sex is risky and there was a need to use condom during anal sexual act.	low risk of bias
Duby et al. 2016 [123, 159]	South Africa; Uganda; Zimbabwe	Qualitative	Purposive sampling; 88 In-depth interviews	Adult women in community (GP)	F: NS	Some participants expressed “shock”, “disbelief”, “disgust”, “embarrassment” and “denial” about anal sex. Some believed that anal sex should not be openly discussed. There were misconceptions that the back side vaginal sex and anal sex were the same sexual act by the participants until it was explained by the researcher with annotated diagram. A number of participants preferred to use alternative name for anal sex e.g. “hidden part”, “down there” and “where faeces comes out”. Women reported engaging in anal sex in order to satisfy their partners’ sexual pleasure/ They considered anal sex to be safer than vaginal sex and as an alternative sexual act when vaginal sex was not feasible. Some engaged in anal sex for better financial gains.	low risk of bias
Mazeingia et al. 2017 [125]	Ethiopia	Qualitative	Snow ball Sampling; 18 In-depth interviews	FSW (KAP)	F:18–39	Participants engaged in anal sex with their clients for financial gains, for personal sexual pleasure, due to coercion by clients and to satisfy their lovers (husband or boyfriends).	low risk of bias
Shayo et al. 2017 [120]	Tanzania	Mixed methods	Purposive sampling; 20 Focus group discussions	Adolescent and Adult in the community (GP)	F:> = 15	Heterosexual anal sex was perceived to be more culturally acceptable especially among the younger population and it is regarded as part of love making. Some women said men force them to practice anal sex against their wish while other women engage in anal sex to protect their sexual relationships. Some women suffered discrimination among their peers for refusing anal sex. A number of participants believed that anal sex is safe without using any barrier methods. Lubricants such as “jelly” were believed to enhance anal sex pleasure and to minimise risk of injury.	low risk of bias

*GP* General population, *KAP* Key affected population, *M* Male, *F* Female, *FSW* Female sex worker, *NS* Not stated, *Yrs* Years

The majority of the studies (90 out of 103) focused on participants aged 10 to 49 years (Additional file 2: Table S1). Eleven out of 91 studies included only adolescents (aged ≤19 years) [40, 46–48, 54–56, 127]. Seven studies did not indicate the study population age [35, 81, 100–102]. Overall, 46 studies included both male and females [35, 37–48, 51–56, 59, 64–66, 68, 70, 73, 78, 80, 84, 87, 92, 94, 101, 102, 111, 114, 120, 126–129, 134, 136], 51 studies included women/girls only [36, 49, 50, 57, 58, 60–63, 67, 69, 71, 72, 74, 76, 77, 79, 81–83, 85–91, 96–99, 105, 106, 109, 110, 112, 113, 115–119, 121, 132, 133, 135], and eight studies included men/boys only [75, 93, 95, 107, 108, 130, 131, 137].

### Reported condom use during heterosexual oral and anal sex

Condom use was reported in 19 studies during heterosexual anal sex [38, 39, 52, 53, 56, 59, 61, 62, 73, 75, 77, 79, 84, 87, 90, 106, 112, 113, 115, 120], five studies during oral sex [38, 52, 53, 56, 61], and 29 studies for combined vaginal, oral and anal sexual experience [40, 47, 54, 57, 58, 60, 62, 63, 65, 67–69, 76, 80, 82, 85, 88, 92, 97, 98, 105, 107, 111, 116–119, 135]. (Additional file 2: Table S2).

Four (three from South Africa and one from East Africa) out of six studies that reported condomless anal sex were among key affected populations [53, 77, 87, 90], and one study was a general population study in South Africa [77]. Condomless oral sex was also reported in a study among HIV positive men and women in South Africa [53]. Reported condom use tended to be higher during heterosexual anal sex than during oral sex, and was more frequently reported among key affected populations than general populations during heterosexual anal sex. The range of any condom use during oral sex was 1.7–16.5% among three general population studies [38, 52, 56]. Of these, consistent condom use during oral sex was reported by 13.2% of Nigerian and 16.5% of Zimbabwean students [38], and by 12.2% of high school students in Ethiopia [52]. A study in South Africa showed that 54.8% of FSWs reported consistent condom use during oral sex [61].

The range of any condom use during heterosexual anal sex was 6.7–73.1% among eight general population studies [38, 39, 52, 56, 59, 73, 77, 120]. Consistent condom use during anal sex was reported by 22.5 and 27.5% of Nigerian and Zimbabwean students respectively [38], 26.1% of Ethiopian high school students [52] and 36.4% of adolescents and young adults in Tanzania [120]. Among five key affected population studies, the range of condom use during heterosexual anal sex was 13.2.0–67.0% [61, 75, 79, 84, 112]. Two of these studies reported consistent condom use of 45.0% among Kenyan FSWs with their clients [79] and 50% among HIV positive women in a South African city [61]. The range of condomless heterosexual anal sex among four key affected population studies was 7.0–54.3% [53, 79, 87, 90].

### Prevalence of reported oral sex

**Fig. 2:**
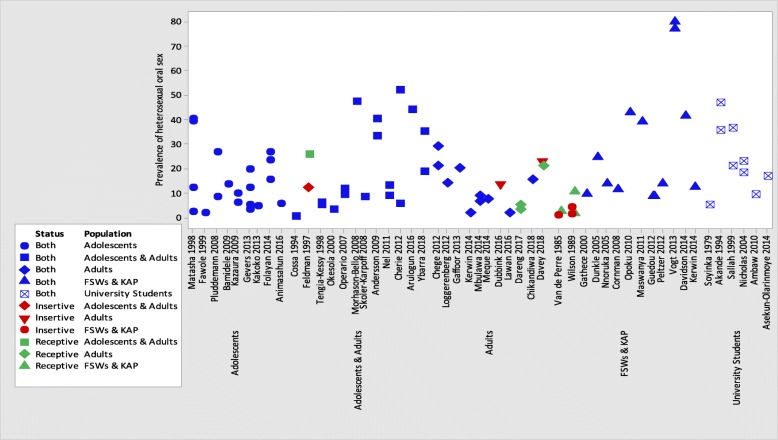
Prevalence of oral sex by study population

Only six (two from South Africa and one each from Nigeria, Rwanda, Zambia and Zimbabwe) out of the 51 oral sex studies described the prevalence of reported oral sex as either giving or receiving oral sex [36, 37, 39, 69, 71, 72]. Most studies that reported on prevalence of oral sex were from South African region, among adolescents and young adults, and key affected populations (Fig. 2, Additional file 2: Table S1 and Additional file 1: Figures S1 and S2).

#### Reported prevalence of ever practiced oral sex

Twenty-eight general population studies reported on the prevalence of ever practising oral sex (Table 1), of which only two studies (Nigeria and Zambia) described the prevalence of giving and receiving oral sex separately [39, 71]. However, the Zambian study combined these data by gender [39]. Of the remaining 26 general population studies reporting oral sex prevalence, 14 described this by gender: five among adolescents and adults [50, 63, 65, 73, 126]; four among adolescents [46, 48, 54, 135]; two studies among adult women [60, 132]; two studies among university students [41, 66]; and one study among an adult men only [130]. Men/boys tended to report a higher prevalence of ever practising oral sex compared to women/girls across these populations. For example, 26.2% of boys reported ever practising oral sex compared to 8.1% of girls in a cross-sectional study in South Africa [46], and 22.8% of males reported ever practising oral sex compared to 18.2% of female South African university students [41]. The range of reported oral sex prevalence in the remaining studies was 5.0–46.4% among university students [35, 38, 64, 68]; 1.7–26.6% among adolescents [47, 55, 56, 127]; 3.0–47.2% among a combined population of adolescents and adults [45, 52, 128, 136], and 1.7–40.8% in three studies among adult populations [130–132]. In all but adult populations, higher prevalences of ever practicing oral sex were recorded after 2000 compared to before 2000. Studies conducted among university students reported a relatively higher prevalence of oral sex compared with other groups within the general population.

Ten studies amongst key affected populations described prevalence of ever practising oral sex [36, 42, 49, 57, 61, 70, 129, 131, 133, 137]. Three studies were among FSWs, of which only a Rwandan study described prevalence of giving and receiving oral sex separately [36, 57, 133]. The Rwandan study showed that 1.8 and 0.5% of FSWs reported that they ever received or gave oral sex respectively [36]. Prevalence of ever practiced oral sex in the other two studies among FSWs were 9.0% in Kenya [133] and 24.1% in South Africa [57]. The prevalences of ever practiced oral sex in two Nigerian studies were reported to be 1.5% among adult men and women in the general community [70] and 13.3% among HIV positive men and women [42]. In Ghana, 42.3% of ‘women considered to be at risk of STIs’ (working in food and recreational facilities) ever practiced oral sex [49]. Three studies from South Africa reported prevalences of ever practising oral sex among key affected population [61, 129, 137]. A study showed that higher proportion of HIV positive men (79.4%) in the community/clinic reported ever practising oral sex than HIV positive women (76.5%) [129] while the two other studies reported prevalences of ever practiced oral sex of 13.9% [57] among HIV positive women [61] and 15.0% among HIV positive adult men [137] in the community.

#### Prevalence of oral sex by other reporting periods

Data on the prevalence of oral sex using other reporting periods came from 11 general population studies [40, 51, 52, 54, 56, 58, 59, 62, 69, 72, 134]. Three of these studies found that men/boys generally reported a higher prevalence of oral sex than in women/girls. For example, any oral sex in the past 3 months was reported by 29.0 and 21.0% of 18–34 year old Kenyan men and women respectively [51], and by 4.8 and 2.8% of 12–15 year old South African boys and girls respectively [54]. In contrast, a Tanzanian study showed that having had oral sex during their first sexual experience was reported by 39.0 and 40.0% of primary school boys and girls, respectively [40]. Among a combined population of adolescents and adults in Addis Ababa, the prevalence of reported oral sex in the past 12 months among the 5.4% of the study population that had ever practiced oral sex was 51.6% [52]. Two South African studies reported the prevalence of oral sex in the past 3 months as 8.0% among girls/women above 16 years [58] and 19.9% among women aged 18 years and above [62]. Reported prevalence of oral sex in the past 6 months ranged between 32.9 and 40.0% among adult men and women with their casual and steady partners, respectively in Soweto [59], 13.4% among women in rural Mopani District [69], and 6.2% of men and 8.7% of women in Cape Town [134]. Finally, 22.8 and 21.0% of pregnant and postpartum women in Cape Town described either giving or receiving oral sex in the past 12 months respectively respectively [72].

Among four key affected population studies, two studies among HIV positive men and women described the prevalence of reported oral sex in the past 3 months to be 11.0% in KwaZulu-Natal and 13.4% in Mpumalanga in South Africa [44, 53]. A third study among FSWs and their clients in Harare, Zimbabwe, described higher reporting by men of receiving oral sex from FSWs during their last sexual act than by FSWs giving oral sex during their last sexual encounters (10.0% vs 1.0%) [37]. In the same study, 4.0 and 1.0% of men and FSWs respectively reported giving/receiving oral sex during their last sexual act [37]. Another study among FSWs from South Africa, Uganda and Benin reported that the prevalence of oral sex in the past month with clients was 8.3% [67].

### Factors associated with engaging in oral sex

Eight studies investigated factors associated with reported oral sex (Table 2). Five of these studies reported unadjusted estimates [48, 51, 54, 56, 133]. In 2000, a study in Kenya found that older FSWs were less likely to have ever engaged in oral sex than younger FSW [133]. In 2012, another study in Kenya, showed that men tended to report oral sex in the past 3 months more than women (29.0% vs 21.0%) [51]. Similarly, adolescent boys in Tanzania were more likely to report ever practicing oral sex compared with adolescent girls (9.4% vs 5.8%) [48]. In Nigeria, girls were more likely to report oral sex as their last sexual act compared with boys (23.5% vs 15.1%) [56]. Girls in South Africa aged 12–15 years who were “currently dating” compared to girls that were not currently dating were more likely to have reported ever having oral sex with their partners (8.1% versus 0.6%) and also having oral sex in the last 3 months (6.5% versus 0%,) [54]. Similar results on dating status were also reported among boys (20.2% versus 8.1%) [54].

**Table 2:** Factors reported to be associated with engaging in heterosexual oral and anal sex among adolescents and adults in sub-Saharan Africa

Author; Year; Country	Study population (no of individuals)	Test of association	Reported associated risk factor for Oral sex	Summary of results
Gathece et al. 2000; Kenya [133]	FSW (322)	Unadjusted, Chi Square	Age	Older respondents were more likely to engage in oral sex (X^2^ = 18.847, *p* = 0.002)
Kazaura et al. 2009; Tanzania [48]	Adolescents in schools/community (885)	Unadjusted, Chi Square	gender	Male vs female (9.4% vs 5.8% *p* = 0.07)
Chege et al. 2012; Kenya [51]	Adult men and women in community (846)	Unadjusted, Chi Square	gender	Male vs female (29% vs 21%, *p* = 0.03)
Gevers et al. 2013; South Africa [54]	Adolescents in schools/community (474)	Unadjusted, Chi Square and Fisher’s Exact Test	type of sexual relationship by gender	**For Girls** In the past 3 months: currently dating vs not currently dating (6.5% vs 0%; *p* < 0.01) Ever practiced: currently dating vs not currently dating (8.1% vs 0.6%; *p* < 0.01) **For Boys** In the past 3 months: currently dating vs not currently dating (7.8% vs 2.0%; *p* < 0.06 Ever practiced: currently dating vs not currently dating (20.2% vs 8.1%; *p* = 0.01)
Folayan et al. 2014; Nigeria [56]	Adolescents in schools/community (357)	Unadjusted, Chi Square	gender	female vs male (23.5% vs 15.1%, p = 0.01)
Ambaw et al. 2010; Ethiopia [66]	University students (1945)	Adjusted, Logistic regression	Gender; level of education; faculty; place of residence; marital status	Male (AOR = 1.6, 95%CI1.02–2.57); protestant (AOR = 0.59, 95%CI 0.39–0.9); year one student (AOR = 2.14, 95%CI1.23–3.66); business and economics faculty (AOR = 5.47, 95%CI3.09–9.67), technology faculty (AOR = 6.23, 95%CI3.32–11.67), humanities faculty (AOR = 3.15, 95%CI1.92–5.18), social sciences faculty (AOR = 2.96, 95%CI1.61–5.42) and education faculty (AOR = 3.91, 95%CI2.01–7.61); out of campus (AOR = 1.85, 95%CI1.09–3.14); have boy/girlfriend (AOR = 1.81, 95%CI1.17–2.8)
Cherie et al. 2012; Ethiopia [52]	Adolescents in schools/community (3543)	Adjusted, Logistic regression	age; gender; attitude to oral sex; aspiration for college education; self-esteem; maternal education; partner education; perception of peer oral sexual activity and living arrangement	Younger age (AOR = 3.2, 95%CI 1.9–5.3); female (AOR = 1.3, 95%CI 1.1–2.2); positive attitude to oral sex (AOR = 2.3, 95%CI 1.7–4.5); low aspiration to attend college education (AOR = 3.1, 95%CI = 2.8–5.9); low self-esteem (AOR = 2.1, 95%CI 1.7–3.9); illiterate mother (AOR = 11.5, 95%CI 6.4–18.5); illiterate father (AOR = 1.4, 95%CI 0.9–3.2); friends that engage in oral sex (AOR = 5.7, 95%CI 3.6–11.2); living with both parents (AOR = 0.4, 95%CI 0.2–0.9)
Kerwin et al. 2014; Malawi [131]	Adult men in community (2753)	Adjusted, Logistic regression	no of lifetime sex partners; ever used condom for oral sex; history of spending in the past 3 months	higher total lifetime sex partner (AOR = 1.04, 95%CI 1.02–1.06); ever used condom (AOR = 3.16, 95%CI 1.47–6.8); history of spending in the last 3 months (AOR = 1.94, 95%CI 1.55–2.42)
Author; Year; Country	Study population (no of individuals)	Test of association	Reported associated risk factor for Anal sex	Summary of results
Chege et al. 2012; Kenya [51]	Adult men and women in community (846)	Unadjusted, Chi Square	Gender	female vs male (25% vs 16%, p = 0.03)
Gevers et al. 2013; South Africa [54]	Adolescents in schools/community (474)	Unadjusted, Chi Square and Fisher’s Exact Test	type of sexual relationship by gender	**For Girls** In the past 3 months: currently dating vs not currently dating (2.4% vs 0%, *p* = 0.09) Ever practiced: currently dating vs not currently dating (3.3% vs 0%, *p* = 0.04) **For Boys** In the past 3 months: currently dating vs not currently dating (13.3% vs 2.0%, p < 0.01) Ever practiced: currently dating vs not currently dating (15.6% vs 6.0%, p = 0.01)
Priddy 2011; Kenya [89]	FSW (200)	Unadjusted, Chi Square	type of sexual relationship	regular vs casual vs primary (35% vs 29% vs 9%, p < 0.01)
Kalichman et al. 2009; South Africa [84]	Adult men and women in community /clinic (M = 2593; F = 1818)	Adjusted, Logistic regression	age; sexual relationship; history of condom use; history of STI; transactional sex; alcohol and cannabis abuse use in past 3 months; HIV test and status; number of sexual partners	Older age (AOR = 0.97, 95%CI 0.96–0.98); married/living with partner (AOR = 0.62, 0.5–0.77); never used condom (AOR = 1.79, 1.49–2.17); history of STI (AOR = 1.64, 1.46–1.85); received gift for sex (AOR = 1.77, 1.57–1.99); given gift for sex (AOR = 1.7, 1.51–1.9); alcohol use in past 3 months (AOR = 2.16, 1.77–2.63); cannabis use in past 3 months (AOR = 1.97, 1.62–2.4); had HIV test (AOR = 1.44, 1.19–1.73); test HIV positive (AOR = 2.62, 1.89–3.63); increased number of sexual partners (AOR = 1.26, 1.2–1.32); unprotected vaginal intercourse (AOR = 0.97, 0.94–0.98); previous vaginal intercourse with condom (AOR = 1.04, 1.03–1.05); increasing percentage condom use during vaginal intercourse (AOR = 5.61, 4.27–7.37)
Ambaw et al. 2010; Ethiopia [66]	University students (1921)	Adjusted, Logistic regression	Faculty; marital status	Business and economics faculty (AOR = 6.3, 95%CI2.64–15.05), technology faculty (AOR = 7.5, 95%CI2.96–18.99), humanities faculty (AOR = 4.59, 95%CI2.19–10.05), social sciences law faculty (AOR = 3.02, 95%CI1.15–7.94) and education faculty (AOR = 5.85, 95%CI2.26–15.1); ever married (AOR = 4.06, 95%CI1.53–10.79)
Veldhuijzen et al. 2011; Rwanda and Kenya [90]	FSW Kigal = 800; Momb = 820	Adjusted, Logistic regression	inconsistent condom use; number of sexual partner; alcohol use before sex; year of sex work and history of genital symptoms	**For the Kigali cohorts** - inconsistent condom use with casual partner (AOR = 5.9, 95%CI 1.4–24.7); had more than 5 sexual partners in last week (AOR = 4.34, 95%CI 1.52–12.36); regular use of alcohol before sex (AOR = 2.83, 95%CI 1.37–5.84). **For Mombasa cohorts** - more than 5 years of sex work (AOR = 2.44, 95%CI 1.22–4.89); inconsistent condom use with casual partner or client (AOR = 2.1, 95%CI 1.10–4.20); condom not used in the last sex (AOR = 3.40, 95%CI 1.70–6.80); had more than 5 sexual partners in last week (AOR = 2.20, 95%CI 1.10–4.30); had genital symptoms (AOR = 3.60, 95%CI 1.70–7.90).
Kalichman et al. 2011; South Africa [87]	Men and women attending bar/night clubs (4965)	Adjusted, Logistic regression	age; education; type of sexual relationship; meeting sexual partner in Shebeen in past months	Age (AOR = 0.97, 95%CI 0.96–0.98); primary sexual partner (AOR = 1.56, 1.19–2.05); casual sexual partner (AOR = 2.33, 1.92–2.83); meeting sexual partner in Shebeen (drinking spot) in the past month (AOR = 1.81, 95%CI 1.47–2.22)
Cherie et al. 2012; Ethiopia [52]	Adolescents in schools/community (3543)	Adjusted, Logistic regression	age; gender; attitude to oral sex; aspiration for college education; self-esteem; maternal education; partner education; perception of peer oral/anal sexual activity and living arrangement	Younger age (AOR = 1.7, 95%CI 1.3–3.1), female (AOR = 2.9, 1.6–4.7), having positive attitude towards anal sex (AOR = 6.2, 95%CI 3.8–12.4), having low aspiration for college education (AOR = 4.2, 95%CI 2.8–8.1), having low self-esteem (AOR = 1.6, 95%CI 1.2–3.1); illiterate mother (AOR = 11.6, 95%CI 7.8–19.6); illiterate father (AOR = 7.8, 95%CI 5.3–14.9); friends that engage in oral/anal sex (AOR = 9.7, 95%CI 5.4–17.7); living with both parents (AOR = 0.4, 95%CI 0.2–0.9)

*AOR* Adjusted odds ratio, *CI* Confidence interval, *M* Male, *F* Female

Two studies from Ethiopia and a study from Malawi reported adjusted estimates for the association between potential risk factors and reported oral sex [52, 66, 131]. A study among 3543 adolescents in high schools in Ethiopia showed that reporting oral sex was associated with having an illiterate mother, being younger (15–16 years compared to 17 years and older), being female, having a perception of oral sexual activity in peers, having a positive attitude to oral sex and low self-esteem [52]. Of the 5.4% who reported ever having had oral sex, 13.5% had initiated oral sex before the age of 10 years [52]. Another Ethiopian study found that ever practising oral sex among university students was associated with male gender; being a first year undergraduate; being a student in faculties of business and economics, technology, humanities social sciences and education; living off campus; being Protestant Christian denomination; and having boy/girl friends [66]. A study among Malawian men that reported ever practicing oral sex had three times odds of ever using condoms, two times odds of spending money in the last 3 months and having higher number of lifetime sexual partners than men with no history of oral sex [131].

### Motivations for engaging in oral sex

Only one study reported on motivations for engaging in oral sex. This study was conducted among Ethiopian school boys and girls aged 15–24 years in Addis Ababa [52]. The main motivations reported by participants were preventing pregnancy (95.9%), minimizing risk of HIV acquisition (86.5%), preserving virginity (85.8%) and reducing the risk of STIs (80.4%). Of those having oral sex within the past 12 months, 48.0% had received a gift in exchange for practising oral sex.

### Prevalence of reported heterosexual anal sex

**Fig. 3:**
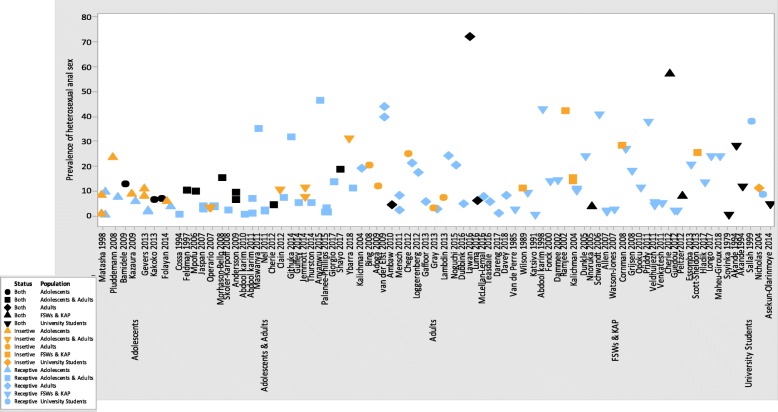
Prevalence of heterosexual anal sex by study population

Sixty-five out of 82 studies distinguished the prevalence of reported anal sex into ‘insertive’ by male participants or ‘receptive’ by female participants [36, 37, 40, 41, 44, 46, 48–51, 54, 56–58, 60–67, 69, 71–77, 79–99, 105–113, 115–119, 121]. Most studies that described anal sex were from Southern and East African countries, among adolescents and young adults, and key affected populations (Fig. 3, Additional file 2: Table S1 and Additional file 1: Figures S4 and S5). We used the same reporting periods as oral sex in presenting prevalences of heterosexual anal sex in different populations.

#### Reported prevalence of ever practiced heterosexual anal sex

Fifty-one out of 82 studies reported on the prevalence of ever practising heterosexual anal sex. Twenty-three out of 51 studies were conducted among key affected populations [36, 42, 49, 57, 61, 70, 74–76, 79, 82, 89, 93, 105–108, 110, 112, 114, 117, 118]. Seventeen of the 28 general population studies described reported prevalence of receptive or insertive heterosexual anal sex separately [41, 46, 48, 50, 54, 60, 63–66, 71, 73, 95–97, 109, 113]. Of these, seven studies were among adolescents and adults [50, 60, 65, 73, 95, 96, 113], five studies were conducted among adolescents only in schools/communities (South Africa [46, 54, 97], Tanzania [48] and Mozambique [63]), three studies among university students in South Africa [41, 64, 66] and two studies among adult women [71, 109]. In seven studies, the proportion of boys that reported ever practising heterosexual insertive anal sex was higher than proportion of girls that reported ever engaging in receptive anal sex [41, 46, 48, 54, 65, 66, 73]. For example, in Tanzania, 8.5 and 5.4% of boys and girls reported ever having insertive or receptive anal sex, respectively [48]. In South Africa, 10.8 and 8.4% of male and female university students reported ever engaging in insertive or receptive anal sex, respectively [41]. In the three studies that were conducted among girls/women only, the reported prevalence of receptive anal sex among adolescents and adults was 34.8% in Tanzania [50] and 7.7–11.3% in South Africa [95], and in a study from South Africa a prevalence of 1.5–1.9% was reported among adult women [60].

In the remaining 11 general population studies that reported prevalence of ever practiced anal sex among boys/men and girls/women: three were among university students [35, 38, 68] and four studies each were among adolescents and adults in schools/community [39, 45, 52, 78] or adolescents in schools [47, 55, 56, 80]. The reported prevalence of ever practising anal sex among university students in Nigeria ranged between 0.3–4.4% [35, 68]. In another similar study, 35.2% among university students in Zimbabwe, and 46.4% among university students in Nigeria reported ever practising in anal sex [38]. Studies that combined adolescent and adult populations reported prevalences between 4.3–34.8% from Zambia, South Africa, Nigeria and Ethiopia [39, 45, 52, 78]. Studies amongst adolescents in schools/communities showed that the prevalence of ever practicing anal sex was 6.7–12.4% in Nigeria [47, 56], 2.4–3.8% in South Africa [80] and 6.4% in Tanzania [55].

In key affected populations, the reported prevalence of ever practising receptive heterosexual anal sex in nine different studies among FSWs was 2.3–42.8% in Rwanda [36], Cameroun [110], Uganda [118], Kenya [74, 79, 89] and South Africa [57, 76, 106]; 42.0% among male truck drivers in South Africa [75]; 25.4% among men attending bars/night clubs in South Africa [93]; 11.5% [49], 2.4% [82] and 0.4% [105] among women working in food and recreational facilities in Ghana, Tanzania and Kenya respectively. Other reported prevalences of anal sex were 3.3–17.1% among HIV positive men and women in Nigeria [42] and South Africa [61], 5.7–72.0% among adult men and women in community/clinics in Nigeria [70] and Cameroun [114], 11.7–20.0% among adult men only in Nigeria [108] and Angola [107], and 13.4% among adolescents and adults women in South Africa [117].

#### Prevalence of heterosexual anal sex by other reporting periods

Six key affected population studies reported specifically on insertive or receptive anal sex within the past 3 months [44, 53, 83, 84, 90, 91]. Higher prevalences of insertive anal sex in the past 3 months were reported by men compared to receptive anal sex reported by women among key affected populations [44, 84]. Similarly, two key affected population studies in South Africa (one study each among HIV positive men and women [44] and adult men and women [84]) showed higher prevalences among men that reported practising insertive anal than women that engaged in receptive anal sex. Three other studies among HIV positive men and women in Kenya, South Africa and Zimbabwe described the prevalence of heterosexual anal sex in the past 3 months to be 18.0% [83], 7.7% [53] and 4.8% [91] respectively.

Four studies among key affected populations presented prevalences of anal sex in the past month [67, 81, 87, 92]. One study compared prevalences of anal sex in the past month by method of data collection and found that women in food and recreational centres in Tanzania reported a higher prevalence of anal sex using daily diaries (2.1%) to record their sexual behaviours compared to face-to-face interviews (1.4%) [81]. The other two studies from South Africa reported the prevalence of insertive anal sex in the past month among men attending bars/night clubs and men patronising alcohol drinking points to be 15.0% [87] and 10.6% respectively [92]. In the same studies, 11.0% of women in bars/night clubs [87] and 6.9% women at alcohol drinking points reported receiving anal sex in the past month [92]. Another study reported that 2.2% of FSWs in South Africa, Uganda and republic of Benin received anal sex in the past month [67]. Other reporting periods used to describe prevalence for anal sex among key affected populations were past 12 months in 3 studies (two from Tanzania and Cote-d’ivoire) [111, 120, 121], and during the most recent sexual acts in 2 studies from Zimbabwe [37] and the Central African Republic [119].

Seven general population studies, including six from South Africa, reported on insertive or receptive anal sex in the past 3 months [51, 54, 58, 62, 77, 98, 99]. The range of reported prevalence of receptive anal sex in three studies among South African women was 5.6–20.3% [62, 77, 98]. Two studies among adolescents and adults reported a prevalence of 2–3.0% [58, 99]. In the same country, a study showed that 1.1% of girls reported receptive anal sex while 7.4% of boys reported insertive anal sex during the same period [54]. A Kenyan study showed that 25.0% men reported insertive anal sex while 21.0% women reported receptive anal sex in the preceding 3 months [51].

Twelve studies used different reporting periods (past 12 months [52, 72, 115], past 6 months [59, 69, 94], past month [85, 86, 88], during first sexual act [40] and last sexual act [56, 116]). Within these reporting periods, the highest reported prevalence of anal sex was 57.1% among anal sex experienced Ethiopian high school students in the past 12 months [52], 9.2% among South African young men and women in the past 6 months [59], 7.8% of South African adult women in the past month [88] and 5.6% of Nigerian boys during their last sexual act [56].

### Factors associated with engaging in anal sex

Eight studies explored factors associated with practising heterosexual anal sex (Table 2). Two of the three studies that presented unadjusted estimates showed that the reported prevalence of heterosexual anal sex was associated with type of sexual relationship [54, 89]. A study amongst South African adolescents found that the prevalence of having ever practised heterosexual anal sex was higher among ‘currently dating’ girls (3.3% vs 0%) and boys (15.6% vs 6.0%) than those with ‘no dating partners’ [54]. A similar result was also described in the same study among boys that reported prevalence of anal sex act in the past 3 months [54]. In Kenya the frequency of anal sex in past month among FSWs was higher among those with regular and casual partners than those with primary partner only [89]. In the same study, the frequency of condom use among FSWs was lower during anal sex than during vaginal sex (data not shown). Unlike the general gender pattern observed in other studies, women in Kenya reported a higher prevalence of heterosexual anal sex than men (25.0% vs 16.0%) [51].

Five studies reported adjusted estimates on factors associated with reported anal sex [52, 66, 84, 87, 90]: A Kenyan study among FSWs showed that the odds of reporting heterosexual anal sex was about four times higher among those with current genital symptoms versus no genital symptoms, three times with inconsistent condom use during last sex compared to those reporting consistent condom use, two times with at least 5 years of sex compared to those with more than 5 years, six times with inconsistence condom use with casual partners than those that used condom, and higher number of sexual partners than those with lower number [90]. A study among FSWs in Rwanda found that inconsistent condom use with casual sex partners (adjusted odds ratio (AOR) = 5.9) and a higher number of sexual partners (AOR = 4.3) were also identified as risk factors for reporting heterosexual anal sex [90]. In addition, the odds ratio for those with regular use of alcohol before sex was about three times associated with reporting anal sex than FSWs that did not regularly use alcohol before sex. A study among men and women attending bars/night clubs in South Africa found the following factors to be associated with reported anal sex in the past month: younger age, having casual sexual partners compared to regular partner, having sex with only one sexual partner compared to having multiple recent sexual partners, and meeting their sexual partners in *shebeens* (alcohol drinking venues) in the past month [87].

Other risk factors associated with engaging heterosexual anal sex that were reported among men and women in the community and special treatment clinics in South Africa included the following [84]: never using condoms; previous transactional sex; cannabis use in the past 3 months; previously tested for HIV and being HIV positive. Being older, married or living with a partner and previous condomless vaginal intercourse reduced the risk of reporting anal sex. Factors associated with ever practising heterosexual anal sex among Ethiopian school boys and girls included younger age, being a boy, having a positive attitude towards anal sex, having low aspirations for college education, having low self-esteem, having a perception of peer engagement in anal sex and having an illiterate mother or father [52]. However, adolescents living with both parents were less likely to engage in anal sex [52]. In another Ethiopian study, reporting ever having had anal sex experience by university students was associated with enrolment in non-medical university faculties compared with students enrolled in a medical faculty [66]. In the same study, university students that had ever married were more likely to report previous anal sex experience than single students [66].

### Motivations for engaging in anal sex

A study in Ethiopia described the motivations for engaging in anal sex among school-attending boys and girls aged 15–24 years [52]. The motivations included minimizing the risk of pregnancy (92.1%), preserving virginity (85.5%), minimising the risk of STIs (82.9%), and minimising risk of acquiring HIV (77.6%). Other motivations reported were desire by the sexual partner, increasing sexual pleasure, and self-preference for anal sex. Amongst the 57.0% who had reported in anal sex in the previous 12 months, 52.3% had received money or gifts for engaging in anal sex.

Four studies (two from Tanzania [103, 104], one from South Africa [100] and one multi-site study from Kenya, Tanzania and Uganda [102]) explored motivations for engaging in anal sex using qualitative research. Their findings showed that motivations differed between men and women (Table 1). Reasons mentioned by women were preserving their virginity, to promote a sexual relationship or to avoid a domestic quarrel, to prevent pregnancy, as an alternative during menstruation or during pregnancy or when there is evidence of a sexually transmitted infection, and in exchange for money [101, 102]. Motivations reported by men included adventure, influence from their peers, to avoid unwanted pregnancy, to enjoy enhanced sexual pleasure and to show sexual supremacy over women [104].

### Cultural meaning, interpretations and personal experiences of anal sex

Twelve qualitative studies explored interpretations of anal sex (Table 1). Five qualitative studies reported on the culture of silence and reluctance to openly discuss heterosexual anal sex [90, 100, 103, 122, 123]. For example, a study in rural South Africa among men and women found that some participants considered heterosexual anal sex to be too sensitive for discussion and some even expressed shock, disappointment and threatened to abandon a focus group discussion when it was brought up for discussion by the facilitator [101]. In the same study, men were reported to be more willing to discuss anal sex than women. In another study in Kenya, men in two counties reported that they were reluctant to discuss anal sex among themselves as it was regarded as a cultural taboo to claim knowledge of, or practice, anal sex in their community [102].

Two studies (Rwanda and South Africa) reported that women, including FSWs, perceived anal sex as punishment, and they only engaged in it to avoid quarrel from their partners/clients and for financial benefits [90, 100]. Other reasons mentioned by participants for engaging in anal sex included adventure, and coercion [100, 120, 124, 125]. There was some evidence of misunderstanding the definition of anal sex; for example, one South African study reported that participants in rural part of Soweto believed anal sex to mean “penile-vaginal penetration from behind” [100]. In several other studies, interviewees believed that anal sex is “foreign” to the African culture [66, 90, 101] or that it is exclusively practiced by men who have sex with men [101]. Some believed anal sex is safer than penile-vaginal sex [120, 124].

Seven qualitative studies presented personal experiences of men and women about heterosexual anal sex [90, 100, 101, 103, 104, 120, 124]. Findings from three studies showed that men expressed more desire for anal sex than women as they often regarded the act as a sign of manhood, and women were reported not to be receptive to openly discussing or demanding anal sex [100, 103, 124]. Tanzanian and South African studies reported that both men and women sometimes used proxy names such as slang or colloquial terms to describe anal sex [101, 104]. A study among FSWs in Kigali reported extreme resentment towards clients who asked for anal sex as they regarded the practice to be uncomfortable, emotionally painful and associated with STIs and faecal and urinary incontinence [90]. However, some young women in Tanzania said that anal sex was more acceptable and enjoyable when performed with “jelly” lubricants [120].

### Assessment of quality of studies

The detailed assessment of risk of bias are presented in Additional file 2: Table S3 and Additional file 1: Figures S3 and S6. Overall, 53 out of 94 quantitative studies assessed had low risk of bias in their methods: 51 of these described heterosexual anal sex [40, 52, 54–57, 59, 60, 62, 64–67, 69–76, 79, 80, 82–84, 86–88, 90–93, 95, 96, 98, 99, 107–112, 115–121] while 24 studies described oral sex [40, 52, 54–60, 62, 64–67, 69–73, 131, 132, 135–137]. The majority of articles assessed to be low risk were from Southern and Eastern Africa. Most studies with a high risk of bias used convenience sampling techniques to recruit study participants, had unclear eligibility criteria, did not include operational definition of outcome measure in the methods, control for potential confounders and present prevalence of oral or anal sex by the reported sexual behaviour role, and gave no indication as to whether ethical approvals were obtained (data not shown – Additional file 2: Table S3). All the nine qualitative studies assessed had low risk of bias (Additional file 2: Table S4).

## Discussion

This is the first systematic review of reported prevalence of oral and anal sex among adolescents and adults reporting heterosexual sex in SSA. The review showed a large range of prevalences for both behaviours. Generally, the range of prevalences were similar among key affected and general populations. However, reported prevalences of oral and anal sex among FSWs and university students tended to be higher than other population groups of adolescents and adults. In addition, reported prevalences of both oral and anal sex tended to be higher among males than females. Few studies reported the use of condoms or other barrier methods with oral and anal sex with a number of them reporting low or inconsistent condom use during oral and anal sex. Factors associated with these behaviours showed that those who engaged in oral and anal sex often also engaged in other high-risk activities such as having frequent and multiple sexual partners, illegal substance or alcohol use, and inconsistent condom use. Oral and anal sex are important modes of transmission for STIs, and these data are vital for understanding sexual behaviours in populations at high risk for STIs including HIV, oral and anal cancers.

Findings from three qualitative studies provided possible explanations for engaging in unprotected anal sex [100, 102, 103] including that it was regarded as less risky than vaginal sex, and could prevent STIs including HIV. Anal sex was also associated with exchange of money or gifts among FSWs. Studies from USA also showed that the belief that oral sex is without health risk, and not regarding oral and anal sex as “having sex” might have accounted for low condom use [138, 139]. Qualitative research also illustrated a culture of silence in discussing anal sex, and many of those that discussed it expressed shock and disbelief that such sexual act existed within heterosexual relationships. This, combined with the belief by some boys/men that performing heterosexual anal sex demonstrates a supremacy over girls/women, may further create a culture of secrecy, shame, and transgression. Indeed, women reported coercion for anal sex and a perception that men engaged in it to punish them. In such an environment, discussion about safety and condom use may be undermined, and strategies for STI control may be challenging. More research is needed to further understand motivations for and meaning of engaging in oral and anal sex in SSA, as well as opportunities and challenges for addressing violence, safety and STI/HIV risk.

The comparatively higher prevalences of oral and anal sex among some key affected populations compared to general populations in this review has also been widely reported in high income countries. Some studies among key affected populations suggested that these behaviours were associated with increased use of alcohol and substance abuse during sexual activity, increased frequency of sexual acts and multiple sexual partners [140–144]. Men who engaged in anal sex with FSWs were more likely to have consumed alcohol and be a frequent customer [140]. Apart from these reasons, it has also been reported that FSWs engaged more in oral and anal sex to satisfy their clients’ requests and for financial gain [140, 145]. This was corroborated by two qualitative studies in this review where FSWs reported economic gain as the main reason for engaging in anal sex [90, 103]. Adolescents in Ethiopia also reported receiving gifts or money for practicing oral and anal sex [52].

We observed high prevalences of oral and anal sex among university students, and this is similar to finding from studies in high-income countries. In the UK NATSAL survey, young people were more likely to report oral and anal sex than older adults, irrespective of gender [21]. The increased reporting of oral and anal sex among young people has been associated with changing perception of sexual activity among younger generation and the influence of social media including pornography amongst others [146–149]. An Australian study that was conducted among young people found that previous anal sex experience was associated with frequent use of pornography [149]. It will be important that adolescent health providers are aware that these behaviours are now being practiced by young people across the continent and education programmes will need to be tailored to addressing the risk associated with these behaviours and how these can be prevented.

Although routine testing for oropharyngeal and anal infections is recommended in high-income countries for sexually active MSM [150, 151], some argue that exclusion of sexually active women with history of receptive oral and anal sex from routine testing will lead to missed opportunities for early detection of STI and the prevention of onward transmission [150, 151]. The cost-effectiveness of routine testing for asymptomatic pharyngeal and anal infection is unclear [151]. Routine testing strategy is not yet a feasible option for people reporting heterosexual oral and anal sex in SSA. However, raising awareness on the risk of STIs during unprotected oral and anal sex through information, education and counselling programmes could be a practicable strategy in the region. It is also imperative that policy makers in the region expand the concept of hetero-normative sexual act to include oral and anal sex.

The higher prevalence of reported oral and anal sex by males in this review should be interpreted within a context of reporting bias. Studies in SSA and other regions have shown that during self-reported sexual behaviour interviews, males tend to report a higher number of sexual partners, non-marital partners and concurrent relationships than females, and females may under-report numbers of sexual partnerships [152–154]. Reporting differences by gender may also vary according to the specific sexual behaviour. Available evidence from population studies in UK and USA showed that more men or boys reported receiving oral sex than women or girls in heterosexual relationship [21, 155]. In the same report, more girls were reported to give oral sex than boys. Some researchers argued that gender differences in the reporting of sexual activity might be influenced by the perception of sexual pleasure, health risks and beliefs [156, 157]. A qualitative study further showed that men preferred to receive oral sex from their partners than to give their partners because they perceived receiving oral sex to be less risky and giving oral sex to be a dirty and dangerous practice [158].

The strengths of this review are the range of prevalences across geographic sub-regions, populations and ages within SSA. However there are a number of limitations. There were very few population-based estimates of the oral and anal sex prevalence such as prevalences reported in the population-based studies in the UK and Australia [18, 21]. Instead, there was a wide range of prevalences of oral and anal sex observed in various population sub-groups which are unlikely to be generalizable. In addition, there were several methodological weaknesses in the studies reviewed including consistent operational definitions of oral and anal sex. Furthermore, studies also used different reporting periods making it challenging to pool and compare results across population sub-groups and settings in SSA.

In addition to reporting gender reporting bias discussed above, there is likely to be under-reporting of these sensitive behaviours due to social desirability bias, especially for heterosexual anal sex, since this practice is not well accepted in some communities [101, 102, 159]. Many studies were assessed as having a high-risk of bias and did not provide information on how their sample sizes were determined. Lastly, restricting our inclusion criteria to only published peer reviewed articles, limiting our search to seven databases and exclusion of MSM that also engaged in heterosexual oral and anal sex could have missed other studies that reported on oral and anal sex in the region.

## Conclusion

In summary, oral and anal sex are commonly practiced among adolescents and adults reporting heterosexual sex in SSA, often without condom use. Future sexual reproductive health research investigating risks for STIs should incorporate questions on oral and anal sex using clear definitions of these behaviours. Well powered and rigorous population based study designs similar to studies in United Kingdom and Australia [18, 21, 160] are needed to understand population estimates of these behaviours, their associated morbidities, and changes in sexual behaviour trends over time. Researchers should also consider using qualitative research methods, complimentary tools such as pictures/drawings and other visual aids to elicit more accurate responses from participants [159]. Accurate data are needed to inform reproductive and sexual health policies, and information on oral and anal sex and their health risks should be included in information, education and counselling messages for both the key affected and general populations.

## Additional files

Additional file 1: **Figure S1.** Prevalence of oral sex by sub-region. **Figure S2.** Prevalence of oral sex by population category. **Figure S3.** Prevalence of oral sex by risk of bias. **Figure S4.** Prevalence of anal sex by sub-region. **Figure S5** Prevalence of anal sex by population category. **Figure S6.** Prevalence of anal sex by risk of bias. (DOCX 122 kb)

Additional file 2: **Table S1.** Selected data from quantitative studies reporting on heterosexual oral and anal sex in sub-Saharan Africa by year of publication. **Table S2.** Reported condom use during penetrative heterosexual sex (oral, anal and vaginal). **Table S3.** Risk of bias assessment for quantitative studies. **Table S4.** Risk of bias assessment of qualitative studies. (XLSX 41 kb)
