# Supplementary material for: Reported oral and anal sex among adolescents and adults reporting heterosexual sex in sub-Saharan Africa: a systematic review
Source: Reprod Health. 2019 May 6;16:48. doi: 10.1186/s12978-019-0722-9 (PMC6501425; doi:10.1186/s12978-019-0722-9)
Supplement: Supplementary file 1 — Figure S1. Prevalence of oral sex by sub-region. Figure S2. Prevalence of oral sex by population category. Figure S3. Prevalence of oral sex by risk of bias. Figure S4. Prevalence of anal sex by sub-region. Figure S5 Prevalence of anal sex by population category. Figure S6. Prevalence of anal sex by risk of bias. (DOCX 122 kb) [file 12978_2019_722_MOESM1_ESM.docx]

***Figure S1: Prevalence of oral sex by sub-region***

*Multiple data points for author and year in the same study by:* ***Educational level – primary & secondary -*** *(Matasha et al 1998);* ***Gender – male & female -***  *(Wilson et al 1989; Matasha et al 1998; Tengia-Kessy et al 1998; Nicholas et al 2004; Operario et al 2007; Pluddemann et al 2008; Kazaura et al 2009; Chege et al 2012; Gever et al 2013; Vogt et al 2013; and Folayan et al 2014); Mbulawa et al 2014; Ybara et al 2018;* ***Range value - the reported range of prevalence -*** *(Kerwin et al 2014);* ***Reporting periods – used more than one reporting periods -*** *(Gever et al 2013; Cherie et al 2012 and Folayan et al 2014);* ***Sexual partners – casual & steady partners -*** *(Anderson et al 2009);* ***Study sites – multiple study sites -***  *(Akande 1994; Nell et al 2011); Okafor et al 2005 was excluded in this graph because it presented combined prevalence of oral and anal sex as a single estimate point. FSWs: Female sex workers; KAP: Key affected population. Status – Type of oral sexual practice*

***Figure S2****:* ***Prevalence of oral sex by population category***

*Multiple data points for author and year in the same study by:* ***Educational level – primary & secondary -*** *(Matasha et al 1998);* ***Gender – male & female -***  *(Wilson et al 1989; Matasha et al 1998; Tengia-Kessy et al 1998; Nicholas et al 2004; Operario et al 2007; Pluddemann et al 2008; Kazaura et al 2009; Chege et al 2012; Gever et al 2013; Vogt et al 2013; and Folayan et al 2014); Mbulawa et al 2014; Ybara et al 2018;* ***Range value - the reported range of prevalence -*** *(Kerwin et al 2014);* ***Reporting periods – used more than one reporting periods -*** *(Gever et al 2013; Cherie et al 2012 and Folayan et al 2014);* ***Sexual partners – casual & steady partners -*** *(Anderson et al 2009);* ***Study sites – multiple study sites -***  *(Akande 1994; Nell et al 2011); Okafor et al 2005 was excluded in this graph because it presented combined prevalence of oral and anal sex as a single estimate point. FSWs: Female sex workers; KAP: Key affected population. Status – Type of oral sexual practice.*

***Figure S3***: ***Prevalence of oral sex by risk of bias***

*Multiple data points for the same author and year referred to disaggregated data by:* ***Educational level*** *(Matasha et al 1998);* ***Gender*** *(Wilson et al 1989; Matasha et al 1998; Tengia-Kessy et al 1998; Nicholas et al 2004; Operario et al 2007; Pluddemann et al 2008; Kazaura et al 2009; Chege et al 2012; Gever et al 2013; Vogt et al 2013; and Folayan et al 2014); Mbulawa et al 2014; Ybara et al 2018;* ***Range value*** *(Kerwin et al 2014);* ***Reporting periods*** *(Gever et al 2013; Cherie et al 2012 and Folayan et al 2014);* ***Sexual partners*** *(Anderson et al 2009);* ***Study sites*** *(Akande 1994; Nell et al 2011); A study by Okafor et al 2005 that presented combined prevalence of oral and anal sex as a single estimate point was excluded in this graph. FSWs: Female sex workers; KAP: Key affected Population. Status – Type of oral sexual practice*

***Figure S4: Prevalence of anal sex by sub-region***

*Multiple data points for author and year in the same study by:* ***Data collection methods – Methods of interview used -*** *(Allen et al 2007; van der Est et al 2009; Mensch et al 2011);* ***Educational level – primary & secondary -***  *(Matasha et al 1998);* ***Gender – male & female -*** *(Operario et al 2007; Folayan et al 2014)* ***Study site*** *(Akande 1994; Abdool Karim et al 2010; Veldhuijezn et al 2011; AbdulKarim et al 2011; Nell et al 2011; Palanee-Philips et al 2015);* ***Sexual partners*** *-* ***casual & steady partners -*** *(Anderson et al 2009; Jemmot et al 2014);* ***Reporting period - used more than one reporting periods -*** *(Cherie et al 2012; Gever et al 2013; Folayan et al 2014 Okafor et al 2005 was excluded in this graph because it presented combined prevalence of oral and anal sex as a single estimate point. FSWs: Female sex workers; KAP: Key affected population. Status – Type of anal sexual practice*

***Figure S5: Prevalence of anal sex by population category***

*Multiple data points for author and year in the same study by:* ***Data collection methods – Methods of interview used -*** *(Allen et al 2007; van der Est et al 2009; Mensch et al 2011);* ***Educational level – primary & secondary -***  *(Matasha et al 1998);* ***Gender – male & female -*** *(Operario et al 2007; Folayan et al 2014)* ***Study site*** *(Akande 1994; Abdool Karim et al 2010; Veldhuijezn et al 2011; AbdulKarim et al 2011; Nell et al 2011; Palanee-Philips et al 2015);* ***Sexual partners*** *-* ***casual & steady partners -*** *(Anderson et al 2009; Jemmot et al 2014);* ***Reporting period - used more than one reporting periods -*** *(Cherie et al 2012; Gever et al 2013; Folayan et al 2014 Okafor et al 2005 was excluded in this graph because it presented combined prevalence of oral and anal sex as a single estimate point. FSWs: Female sex workers; KAP: Key affected population. Status – Type of anal sexual practice*

***Figure S6: Prevalence of anal sex by risk of bias***

*Multiple data points for author and year in the same study by:* ***Data collection methods – methods of interview used -*** *(Allen et al 2007; van der Est et al 2009; Mensch et al 2011);* ***Educational level – primary & secondary -***  *(Matasha et al 1998);* ***Gender – male & female -*** *(Operario et al 2007; Folayan et al 2014)* ***Study site*** *(Akande 1994; Abdool Karim et al 2010; Veldhuijezn et al 2011; AbdulKarim et al 2011; Nell et al 2011; Palanee-Philips et al 2015);* ***Sexual partners*** *-* ***casual & steady partners -*** *(Anderson et al 2009; Jemmot et al 2014);* ***Reporting period - used more than one reporting periods -*** *(Cherie et al 2012; Gever et al 2013; Folayan et al 2014 Okafor et al 2005 was excluded in this graph because it presented combined prevalence of oral and anal sex as a single estimate point. FSWs: Female sex workers; KAP: Key affected population. Status – Type of anal sexual practice*
